# Early Silurian chondrichthyans from the Tarim Basin (Xinjiang, China)

**DOI:** 10.1371/journal.pone.0228589

**Published:** 2020-02-13

**Authors:** Plamen S. Andreev, Wenjin Zhao, Nian-Zhong Wang, Moya M. Smith, Qiang Li, Xindong Cui, Min Zhu, Ivan J. Sansom

**Affiliations:** 1 Research Center of Natural History and Culture, Qujing Normal University, Qujing, Yunnan Province, China; 2 Key CAS Laboratory of Vertebrate Evolution and Human Origins, Institute of Vertebrate Paleontology and Paleoanthropology, Chinese Academy of Sciences, Beijing, China; 3 University of Chinese Academy of Sciences, Beijing, China; 4 CAS Center for Excellence in Life and Paleoenvironment, Beijing, China; 5 Department of Earth Sciences, Natural History Museum London, London, England, United Kingdom; 6 Faculty of Dentistry, Oral & Craniofacial Sciences, KCL, London, England, United Kingdom; 7 School of Geography, Earth and Environmental Sciences, University of Birmingham, Edgbaston, Birmingham, England, United Kingdom; Indiana University Bloomington, UNITED STATES

## Abstract

The Sinacanthida ordo nov. and Mongolepidida are spine- and scale-based taxa whose remains encompass some of the earliest reported fossils of chondrichthyan fish. Investigation of fragmentary material from the Early Silurian Tataertag and Ymogantau Formations of the Tarim Basin (Xinjiang Uygur Autonomous Region, China) has revealed a diverse mongolepidid and sinacanthid fauna dominated by mongolepids and sinacanthids in association with abundant dermoskeletal elements of the endemic ‘armoured’ agnathans known as galeaspids.

Micro-computed tomography, scanning electron microscopy and histological sections were used to identify seven mongolepid genera (including *Tielikewatielepis sinensis* gen. et sp. nov., *Xiaohaizilepis liui* gen. et sp. nov. and *Taklamakanolepis asiaticus* gen. et sp. nov.) together with a new chondrichthyan (*Yuanolepis bachunensis* gen. et sp. nov.) with scale crowns consisting of a mongolepid-type atubular dentine (lamellin). Unlike the more elaborate crown architecture of mongolepids, *Yuanolepis* gen. nov. exhibits a single row of crown elements consistent with the condition reported in stem chondrichthyans from the Lower Devonian (e.g. in *Seretolepis*, *Parexus*). The results corroborate previous work by recognising lamellin as the main component of sinacanthid spines and point to corresponding developmental patterns shared across the dermal skeleton of taxa with lamellin and more derived chondrichthyans (e.g. *Doliodus*, *Kathemacanthus*, *Seretolepis* and *Parexus*).

The Tarim mongolepid fauna is inclusive of coeval taxa from the South China Block and accounts for over two-thirds of the species currently attributed to Mongolepidida. This demonstrates considerable overlap between the Tarim and South China components of the Lower Silurian Zhangjiajie Vertebrate Fauna.

## Introduction

Lower Silurian vertebrate assemblages of the Tarim and South China tectonic blocks are dominated by galeaspid agnathans and putative chondrichthyans and are united together within the recently erected Zhangjiajie Vertebrate Fauna [1, 2]. The chondrichthyans are represented by two enigmatic groups, the Sinacanthida ordo nov. and the Mongolepidida [3–7]. Sampling in South China has revealed sinacanthids and mongolepids in Telychian strata of the Xiushan Formation in Guizhou Province (southeast [5, 8]) and similar faunas from the Ymogantau Formation (sensu [9]) on the northwestern margin of the Tarim Basin (Xinjiang Uygur Autonomous Region, northwest [3, 5, 8, 10]). The Xiushan Formation material contains a number of mongolepid and mongolepid-like taxa (*Xinjiangichthys*, *Shiqianolepis*, *Rongolepis* and *Chenolepis*), whilst only *Xinjiangichthys* and Chondrichthyes indet. scales have previously been documented from the Ymogantau [3, 10]. Despite these and other studies on the vertebrate fossils of the Tarim Basin [4, 6, 11–15], a large portion of the material collected from the area in the 1990’s has remained undescribed until now. These new specimens provide supporting evidence for the stratigraphic resolution of the Tarim Basin red beds, an issue that has proved problematic over the last 60 years or so.

The taxa identified here are placed within the total-group Chondrichthyes and add to the burgeoning diversity of Lower Palaeozoic scales that are shark-like in their overall appearance, growth and histology. These include, in approximate stratigraphic order from the Darriwilian (Middle Ordovician) through to the Lower Devonian, *Tantalepis* [16], *Tezakia* and *Canyonlepis* [7, 17], mongolepids [5, 18–20], elegestolepids [20, 21] and *Tuvalepis* [22]. The relationship of these taxa to conventionally defined chondrichthyans (*sensu* [23]) remains contentious despite recent progress in integrating scale-based trees into the phylogenetic framework of early jawed gnathostomes [24]. This comes at a time of renewed evaluation of the chondrichthyan stem, following the introduction of what have previously been regarded as crown-group taxa (e.g. *Doliodus* and *Pucapampella*; [25–29]) into the phylogenetic space occupied by ‘acanthodians’ [30].

### Geological and stratigraphic setting

**Fig 1:**
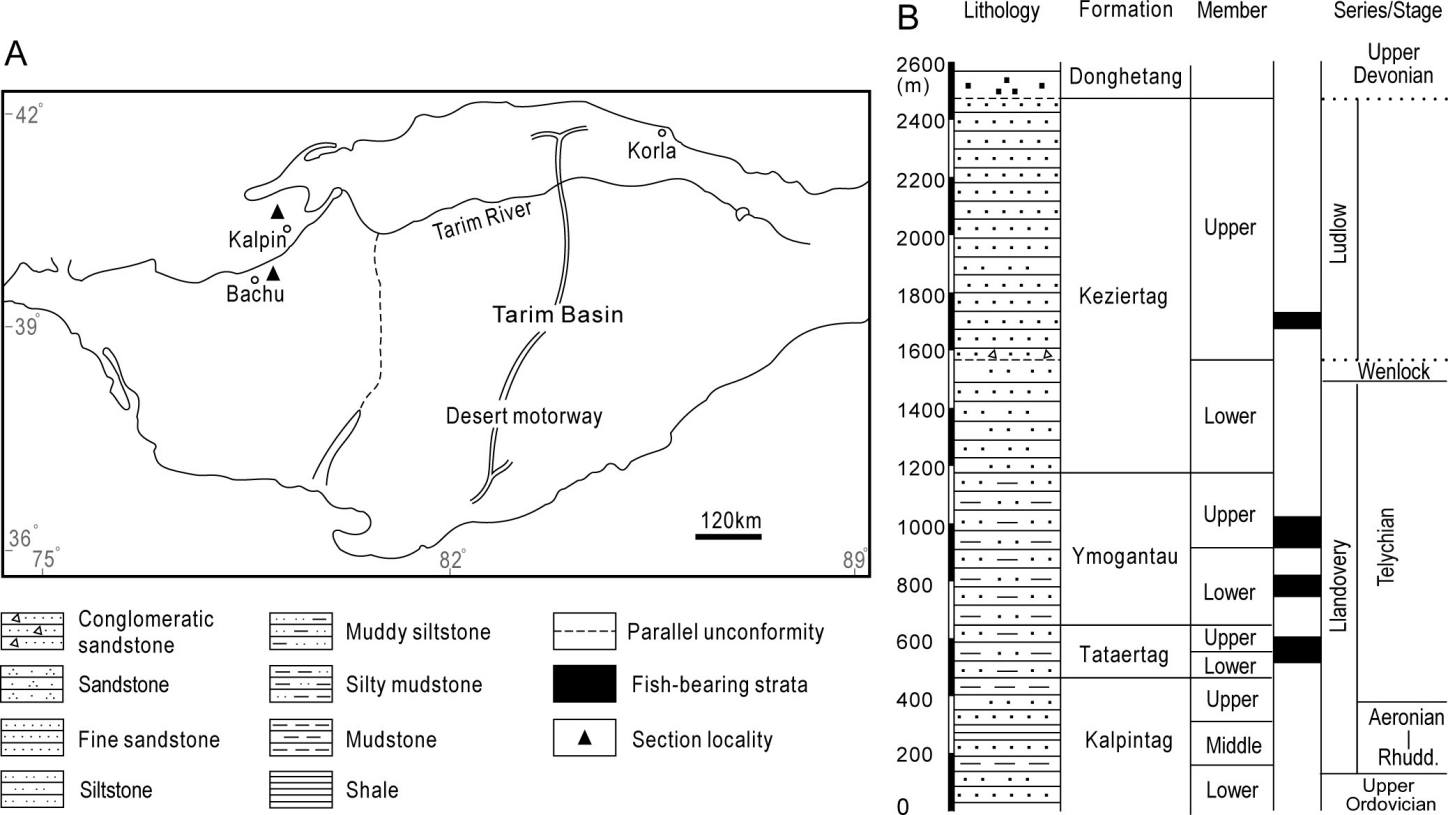
Lower Silurian vertebrate sites and lithostratigraphic Formations in the Tarim Basin (Xinjiang, China) sampled for this study. Map depicting the locations of the sections in Kalpin and Bachu Counties (A) and a summary log of both sections correlated with Silurian chronostratigraphy (data from [3, 8] and this study).

Silurian strata outcropping on the northwestern margin of the Tarim Basin form a continuous depositional sequence reaching approximately 2000m in thickness. Comprised of predominately red sandstones and mudstones, this sequence has been subdivided into four lithostratigraphic units (Fig 1): the Kalpintag, Tataertag, Ymogantau and Kezirtag Formations [9] (synonymous with the previously used Kepintage, Tataaiertage, Yimugantawu and Keziertage in [8, 31]). Of these, the Tataertag and Ymogantau have yielded the abundant fish fossils that are the subject of this study.

### Tataertag Formation (*sensu* [9, 31])

The mostly continental shelf deposits of the Kalpintag Formation grade rapidly upwards into the grey, greyish-white siltstones, sandstones and mudstones intercalated with light-purple, purplish-red siltstones and marlstones of the Tataertag Formation (Fig 1; includes the upper levels of the Kalpintag Formation as defined by [32]). The two members of the Formation (lower and upper) are exposed only in Kalpin County where they are c. 190 m thick [33]. These sequences are thought to represent fluctuating shoreface and neritic environments [31, 32].

Rare invertebrates are represented by gastropods and brachiopods, whereas vertebrate macrofossils are common in the calcareous siltstone beds. A diverse array of fish are known from the Tataertag Formation including the galeaspids *Nanjiangaspis zhangi*, *N*. *kalpinensis*, *Kalpinolepis tarimensis*, *Microphymaspis pani*, *Platycaraspis tianshanensis*, *Hanyangaspis guodingshanensis*, *H*. sp., and the putative chondrichthyan spine genera *Sinacanthus* and *Neoasiacanthus* [8, 10, 34–36]. The presence of these forms has been used to interpret Tataertag as coeval with the early Telychian Rongxi Formation from the middle and lower reaches of the Yangtze River in the South China block [1, 36]. This agrees with Llandovery dates proposed for the Tataertag based upon acritarchs, scolecodonts, cryptospores and plant cuticles [37].

### Ymogantau Formation (*sensu* [9, 31])

The Bachu and Kalpin sections of the overlying Ymogantau Formation (Fig 1) are c. 160 m and 520 m thick respectively, with strata divided into lower and upper members [33]. These consist of purplish-red tuffaceous and argillaceous siltstones and mudstones intercalated with greyish-green tuffaceous fine-grained sandstones and siltstones and are thought to represent predominant tidal flat deposition [31, 32]. Invertebrate macrofossils are rare with only a few lingulid brachiopods (*Lingula* sp.), gastropods and bivalve molluscs found [38]. However, a diverse fossil fish fauna has been described, including the galeaspids *Pseudoduyunaspis bachuensis*, *Hanyangaspis guodingshanensis*, *H*. sp. and the putative chondrichthyans *Sinacanthus wuchangensis*, *S*. *triangulatus*, *Tarimacanthus bachuensis* and *Xinjiangichthys* [8, 36].

Given the absence of age diagnostic taxa, dating of the Ymogantau Formation has been contentious. The main suggestions are as follows: (1) Late Devonian based upon the sedimentologic and tectonic character [39]; (2) Early Devonian based on the conodont *Ozarkodina denckmanni* collected from the lower part of the Ymogantau Formation near the Mukuleke village in Bachu area [40]; (3) middle Aeronian to Wenlock according to the presence of the conodonts *Ozarkodina* cf. *edithae*, *Ozarkodina* sp. A and *Ligonogina silurica* [41]; (4) Telychian based on galeaspid and sinacanthid macrofossils [4, 8, 10] and the presence of the mongolepid *Xinjiangichthys* [3] also reported from the Xiushan Formation in Shiqian, Guizhou Province [5].

Our samples from the Ymogantau Formation contain the conodont *Ozarkodina guizhouensis* together with the mongolepids *Rongolepis cosmetica*, *Shiqianolepis hollandi* and *Chenolepis asketa*. These also occur in the lower Member of Xiushan Formation and suggest that the upper part of the Ymogantau sequence is of middle Telychian age [5, 42, 43] (Fig 1).

## Materials and methods

Studied material from the Kalpin and Bachu Counties (Xinjiang Uygur Autonomous Region) collected in the 1990s with the permission of the IVPP, Chinese Academy of Sciences.

The fish-bearing sections of the Tataertag and Ymogantau Formations crop out in the vicinity of the village Tielikewatie (25 km northwest of Kalpin county town), as well as at the Xiaohaizi reservoir, 20 km northeast of Bachu county town.

Samples were collected by Professors JQ Wang, NZ Wang and other colleagues from the Institute of Vertebrate Paleontology and Paleoanthropology (IVPP), Chinese Academy of Sciences (CAS) in the 1990’s. Disarticulated dermal elements were found in the upper member of the Tataertag Formation and the lower member of the Ymogantau Formation in Kalpin County, and in the lower and upper members of the Ymogantau Formation in Bachu County (Fig 1). Bulk samples were processed in the Key Laboratory of Evolutionary Systematics of Vertebrates, IVPP, CAS, using dilute acetic acid.

X-ray microtomography (μCT) was implemented to examine the inner structure and morphology of individual scales and spines, using the Skyscan 1172 scanner at the School of Dentistry, University of Birmingham (UoB). Specimens were scanned at 59 kV with angular step and range set to 0.3 deg and 192.6 deg respectively. The X-ray beam was attenuated by a 0.5 mm thick aluminum filter. Each scan generated a dataset of 642 radiographs, with pixel size of 1.1μm, 1.24 μm; 1.37 μm and 1.65 μm selected for individual datasets.

The segmentation, volume rendering and virtual sectioning of the tomographic data was performed in Mimics Research 19.0.0.347 3D image processing software.

Specimens were imaged at 2KV and 5kV accelerating voltage with a Hitachi S-3700N scanning election microscope (SEM) at the IVPP, CAS and a Phenom ProX Desktop SEM at the School of Geography, Earth and Environmental Sciences (GEES), UoB.

Doubly polished histological thin sections of scales and spines were investigated via Nomarski differential interference contrast optics with a Zeiss Axioskop Pol polarizing microscope at the GEES, UoB and an Olympus BX51 polarizing microscope at Qujing Normal University. Sections were photographed by microscope-mounted MicroPublisher 5.0 RTV and Olympus DP12 cameras.

Material is deposited at the IVPP, CAS, with accession numbers (designated by an ‘IVPP V’ prefix) being assigned to all figured and some of the imaged non-figured specimens.

## Results

### Systematic paleontology

Class Chondrichthyes Huxley, 1880 [44]

Order Mongolepidida Karatajūtē-Talimaa, Novitskaya, Rozman & Sodov, 1990 [18] sensu [7]

Remarks. The present study follows the most recent revision of the Order in recognising linear odontocomplexes formed by an acellular and atubular dentine (lamellin [18, 45]) as diagnostic for the Mongolepidida [7]. Although not harbouring the tubular system of traditionally-defined dentines [46, 47], lamellin topology and deposition via infill of a pulp-like canal are evidence for its odontogenic origin [45].

Family Mongolepididae Karatajūtė-Talimaa et al., 1990 [18] sensu [7]

Genus *Rongolepis* Sansom, Aldridge and Smith, 2000 [5] sensu [7]

*Rongolepis cosmetica* Sansom, Aldridge and Smith, 2000 [5]

(Fig 2)

2000 *Rongolepis cosmetica* Sansom, Aldridge & Smith, Figs 11 and 12 [5].

2016 *Rongolepis cosmetica* Andreev et al., Figs 3K–3M, 6K, 6L [7].

Holotype. A trunk scale specimen (NIGP 130326) from the Telychian Xiushan Formation, Guizhou Province, China (Sansom et al. 2000 [5], Fig 11h, i).

Material. Five isolated trunk scales (IVPP V 11954.1–5).

Locality and horizon. Lower and upper members of the Ymogantau Formation, Bachu County, Xinjiang Uygur Autonomous Region.

**Fig 2:**
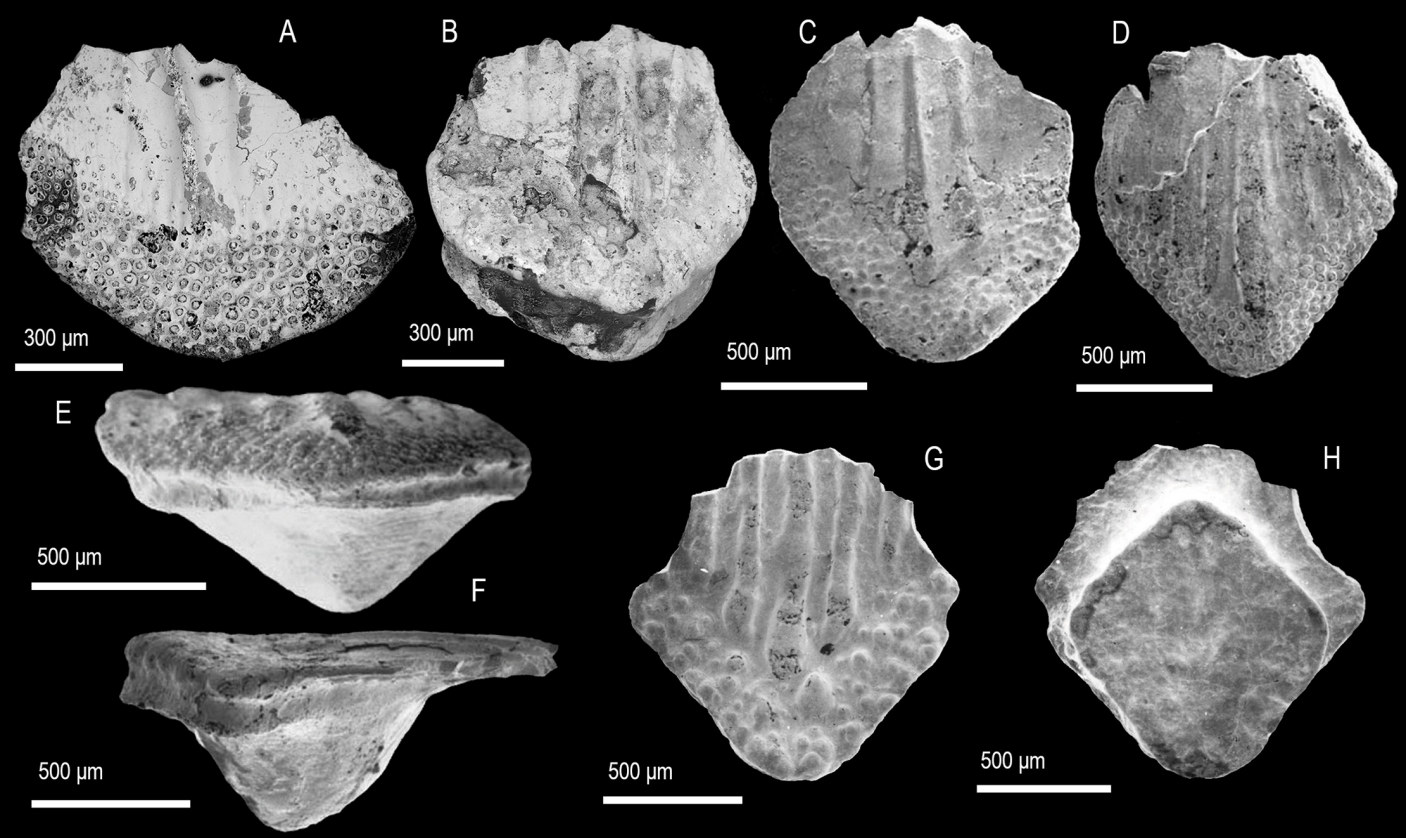
Scale morphology of *Rongolepis cosmetica* Sansom, Aldridge and Smith 2000 [5]. (A) Crown view of a trunk scale (IVPP V 11954.4) with a broad crown. (B) Trunk scale (IVPP V 11954.5) in anterior crown view showing the crown-base junction. (C, F) Trunk scale (IVPPV11954.1) in crown (C) and lateral (F) view exposing the low-profile of the crown. (D, E) Trunk scale (IVPPV11954.2) in crown (D) and anterior view (E). (G, H) Trunk scale (IVPPV11954.3) with coarse tuberculate ornament in crown (G) and base view (H). Anterior to the left in (F) and to the bottom in (A–D), (G, H). SEM micrographs.

### Description

Scales are ovate to trapezoid with a low-profile crown manifesting parallel odontode rows ornamented by a raised medial ridge (Fig 2A–2G). Along its anterior margin the crown develops a crescent-like field of densely packed tubercles that represent the exposed portions of secondary odontodes (Fig 2A–2D, 2E and 2G). The posterior of the lower crown surface is devoid of ornament and can exhibit numerous pores arranged in loose rows. Scale bases are rhombic in outline (Fig 2H) and develop a spur-like central protrusion (Fig 2E and 2F).

Remarks. The material from Xinjiang falls within the range of morphology of *R*. *cosmetica* specimens from their type locality in Guizhou Province, Xiushan Formation [5],[7] and thus *Rongolepis* is retained as monotypic.

Genus *Taklamakanolepis* gen. nov.

Derivation of name. After the Taklamakan Desert of the Tarim Basin, and *lepis*, Greek for scale.

Type species. *Taklamakanolepis asiaticus* gen. et sp. nov.

Diagnosis. As for type and only species.

*Taklamakanolepis asiaticus* gen. et sp. nov.

(Fig 3)

Derivation of name. From the nominative case of the Latin word for Asian, referring to the origin of the species.

**Fig 3:**
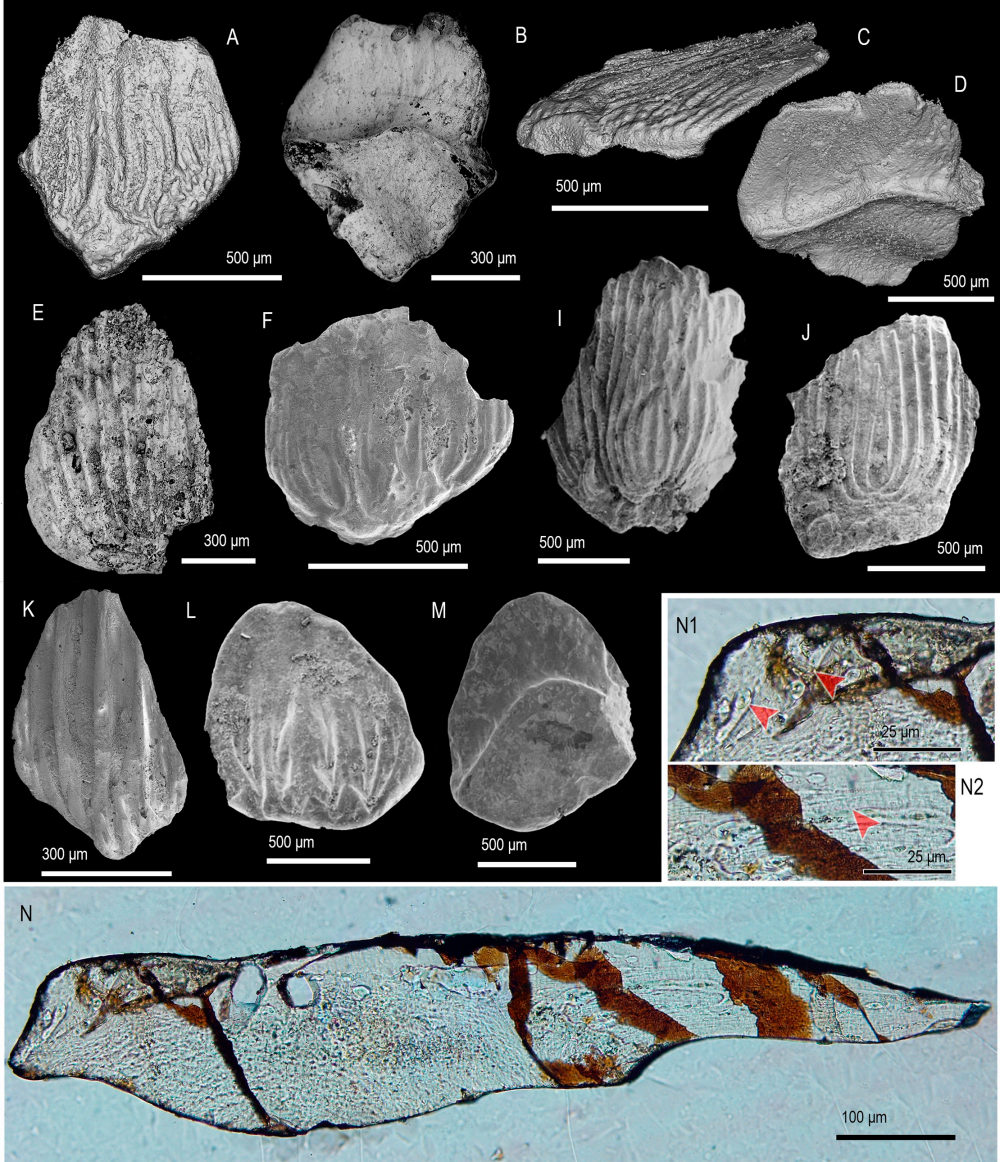
Scale morphology and histology of *Taklamakanolepis asiaticus* gen. et sp. nov. (A–D) Holotype, trunk scale (IVPP V 11952.6) in crown (A), basal (B), lateral (C) and posterior base view (D) revealing the narrow profile and crown architecture of *Taklamakanolepis* gen. nov. scales. (E–L) Crown and (M) base views of scales ((E) IVPP V 11952.7, (F) IVPP V 11952.8, (I) IVPP V 11952.9, (J) IVPP V 11952.10, (K) IVPP V 11952.11, (L, M) IVPP11952.1) demonstrating a range of crown morphologies. (N) Longitudinally sectioned scale (IVPP V 11952.5) showing the structure of the basal bone and the lamellin crown. (N1, N2) Detail views of (N) depicting secondary odontodes at the crown’s anterior and (N2) primary odontodes near the junction with the base. Arrowheads point at contacts between primary or secondary odontodes. Anterior to the left in (C), (N), (N1), N2) and to the bottom in (A), (B), (D–M). SEM micrographs (B, E–M), volume renderings (A, C, D) and Nomarski DIC optics micrographs (N, N1, N2).

Holotype. An isolated trunk scale, IVPP V 11952.6 (Fig 3A–3D).

Material. Fourteen isolated trunk scales and three sectioned trunk scales, including figured specimens (IVPP V 11952.1, 5–11).

Locality and horizon. Lower and upper members of the Ymogantau Formation, Bachu County, Xinjiang Uygur Autonomous Region.

Diagnosis. Mongolepid species possessing scales without a clearly differentiated neck. Crown odontodes overlap in parallel rows and constitute arched elements ornamented with a continuous primary ridge flanked by considerably shallower accessory ridges.

### Description

Morphology. All specimens display trunk scale morphologies characterized by horizontal, low profile crowns with a considerable posterior extension. Crowns are elliptical to ovoid (Fig 3A–3M) and transition into the base without forming a clear neck (Fig 3B). Primary odontodes extend the crown’s length as elongate arched elements with sub-parallel orientation. Odontode ornament exists in the form of a prominent medial ridge and markedly shallower lateral ridges aligned to it (Fig 3A, 3F and 3L). In most specimens the ridges break down into strings of tubercles that become particularly pronounced towards the anterior. A cluster of small lanceolate to irregular secondary odontodes overlaps the ends of primary elements at the anterior crown margin (Fig 3A, 3K, 3L and 3N1). The lower crown surface appears grooved and devoid of canal openings/pores (Fig 3B, 3D and 3M). Scale bases are rhombic with abraded, flat profiles.

Histology. The crown tissue is atubular dentine (lamellin), demonstrating phases of lamellar and globular mineralisation in primary odontodes (Fig 3N, 3N1 and 3N2). Rudiments of pulp canals are seen in the latter but absent from the smaller secondary odontodes. Crown ridges consist of overlapping generations of primary odontodes with the ontogenetically oldest elements occupying an apical position.

The scale bases exhibit acellular bone with a layered structure resulting from fibre-bundle arrangement into apically arched lamellae (Fig 3N).

Remarks. The presence of acellular basal bone in scales with mongolepid type crowns places *Taklamakanolepis* gen. nov. in the Mongolepididae, following [7]. The general appearance of *Taklamakanolepis* gen. nov. specimens is similar to that of the flattened, ovoid scales of *Rongolepis cosmetica* ([5, 7], this study), which are nevertheless distinguished by their tuberculate/polygonal anterior ornament, separation of odontode rows and central protuberance/boss of the base.

Family Shiqianolepidae Sansom, Aldridge and Smith 2000 [5] sensu [7]

Genus *Shiqianolepis* Sansom, Aldridge and Smith 2000 [5]

*Shiqianolepis hollandi* Sansom, Aldridge and Smith 2000 [5]

(Fig 4A–4E)

**Fig 4:**
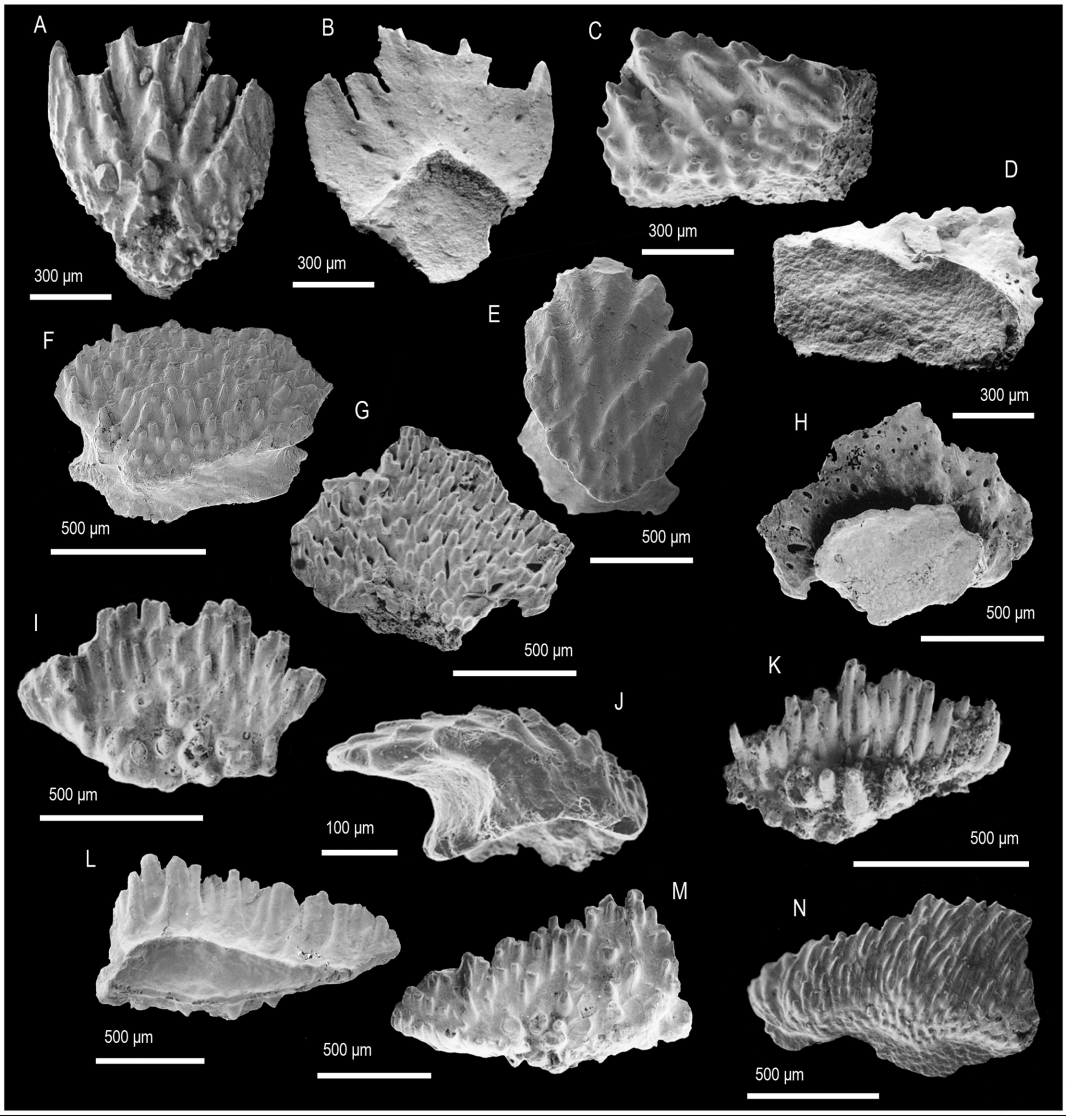
Scale morphology of *Shiqianolepis hollandi* Sansom, Aldridge and Smith 2000 [5] and *Xinjiangichthys pluridentatus* Wang, Zhang, Wang & Zhu 1998 [3]. (A–E) *Shiqianolepis hollandi*. (A, B, E) Two trunk scales, IVPP V 11951 (A, B) and IVPP V 11951.3 (E), with crowns at late stage of development in crown (A, E) and basal view (B). (C, D) Asymmetrical trunk scale (IVPP V 11951.2), with an incipient crown. (F–N) *Xinjiangichthys pluridentatus*. (F–H) Trunk scales with broad crowns, IVPP V 11664.4 (F) and 11664.3 (G, H), in crown (F, G) and basal view (H). (I, J) Trunk scales with compact crowns, IVPP V 11664.5 (I) and IVPP V 11664.6 (J), in crown (I) and lateral view, demonstrating stubby secondary odontodes and posterior curvature. (K–N) Asymmetrical trunk scales, IVPP V 11664.7 (K), IVPP V 11664.8 (L, M) and IVPP V 11664.9 (N) in crown (K, M, N) and basal view (L). Anterior to the right in (J) and towards the bottom in (A–I) and (K–N). SEM micrographs.

**Fig 5:**
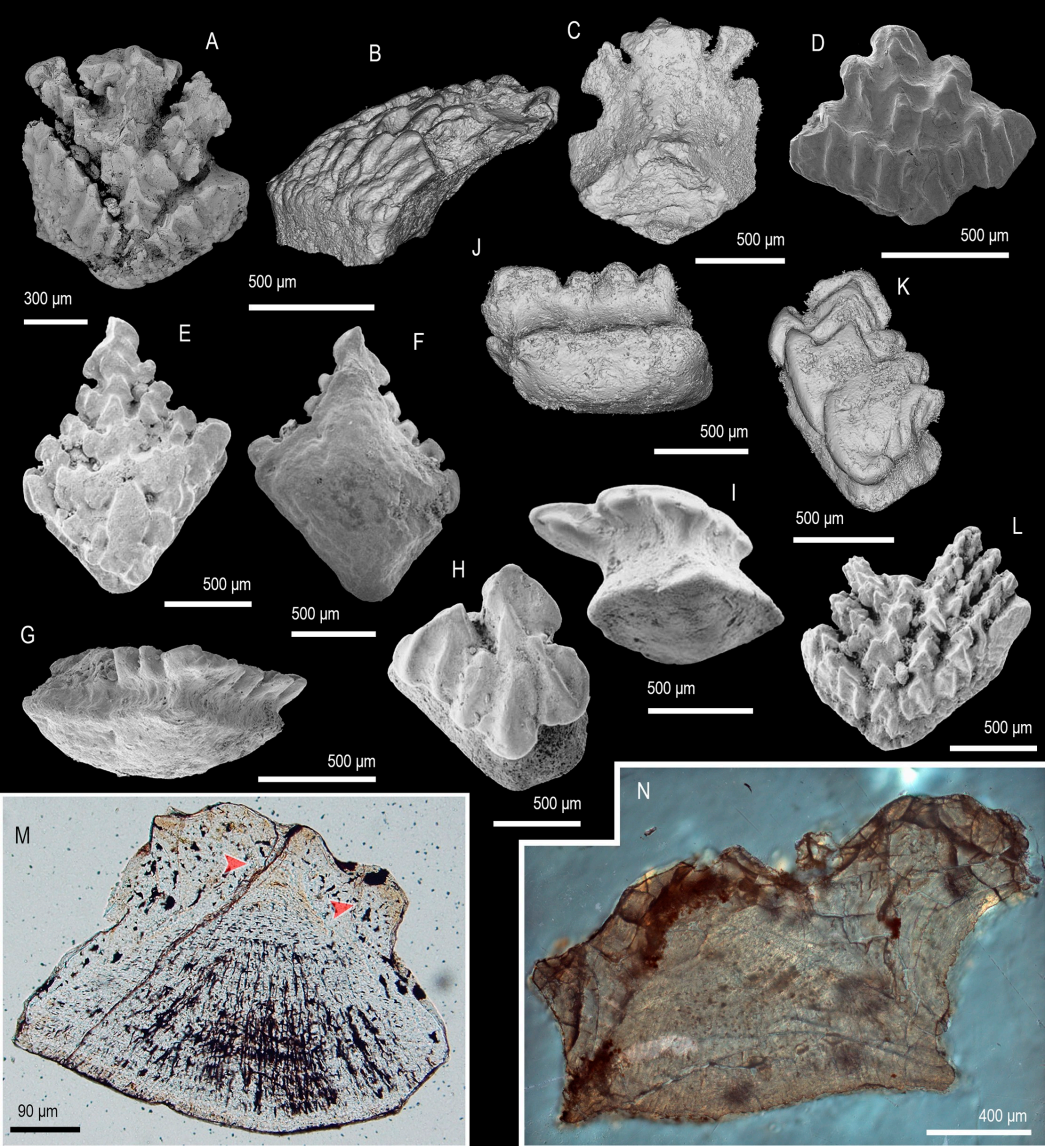
Scale morphology and histology of *Tielikewatielepis sinensis* gen. et sp. nov. (A–C) Trunk scale (IVPP V 11950.10) with an elongated crown, in crown (A), lateral (B) and basal (C) view, holotype. (D) Trunk scale (IVPP V 11950.11) with an incipient crown, in crown (D) view. (E–G) Trunk scale (IVPPV11950.1) with a well-developed base, in crown (E), basal (F) and lateral (G) view. (H–K) Asymmetrical trunk scales, IVPPV11950.5 (H, I) and IVPP V 11950.12 (J, K), with pronounced necks and incipient crowns, in crown (H, K), lateral (I) and basal (J) view. (L) Head scale (IVPPV11950.6) with a low-profile crown in anterior crown view. (M, N) Transverse and longitudinal sections of two trunk scales, IVPP V 11950.13 (M) and IVPP V 11950.15 (N), showing the growth lamellae and fibre spaces of the cellular basal bone and crown architecture. Arrowheads point at contacts between primary odontodes. Anterior to the left in (B), (G), (N) to the right in (I), (J) and towards the bottom in (A), (C), (D–F), (K), (L). SEM micrographs (A, D–I, L), volume renderings (B, C, J, K) and Nomarski DIC optics micrographs (M, N).

**Fig 6:**
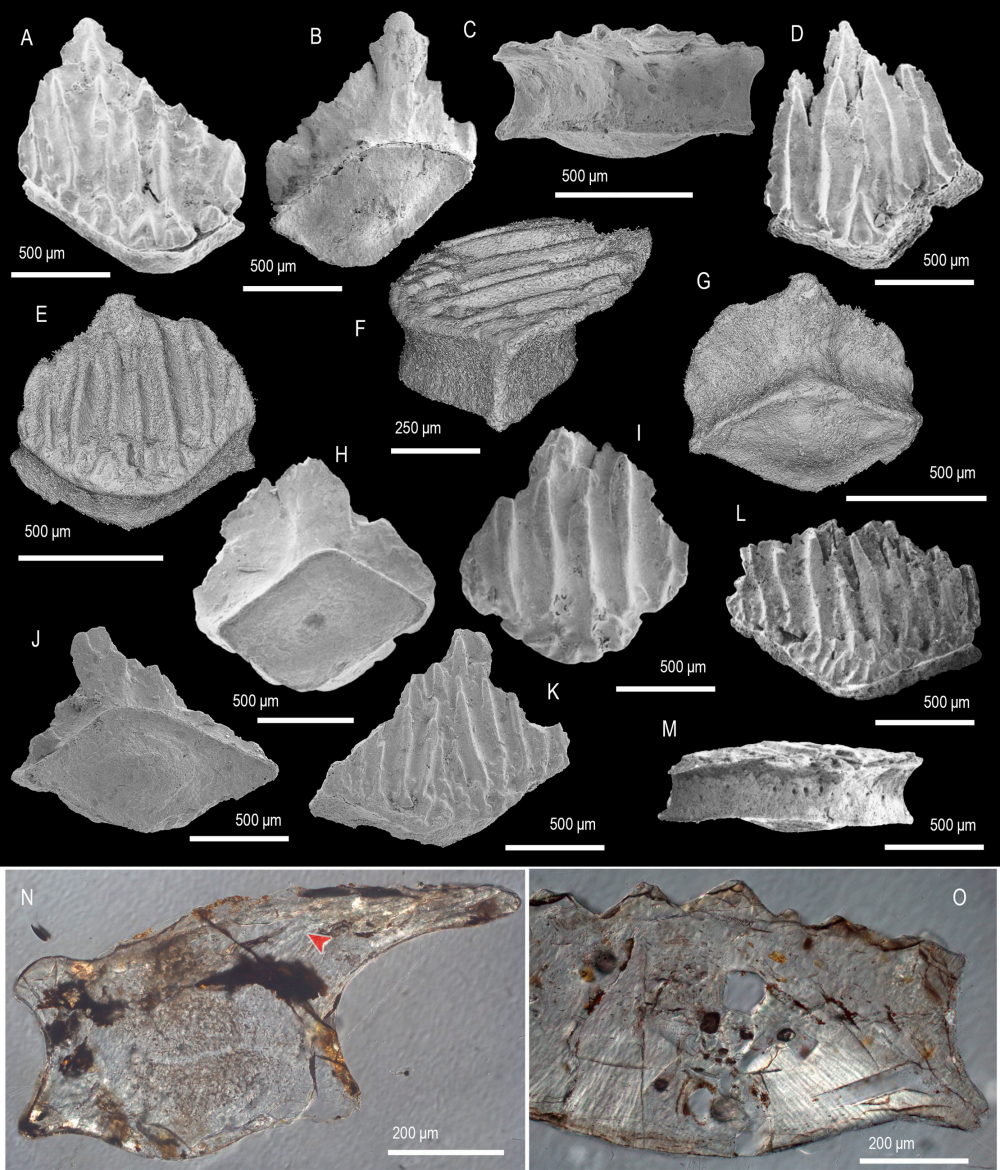
Scale morphology and histology of *Xiaohaizilepis liui* gen. et sp. nov. (A–C) Asymmetrical trunk scale, IVPP V 11949.1, in crown (A), basal (B) and posterior (C) view. (D) Elongate trunk scale (IVPP V 11949.2) in crown view. (E–G) Trunk scale (IVPP V 11949.9) in anterior crown (E), lateral (F) and basal (G) view. (H, I) Trunk scale (IVPP V 11949.3) in basal (H) and crown (I) view, holotype. (J–M) Trunk scales with broad crowns, IVPP V 11949.5 (J, K) and IVPP V 11949.4 (L, M), produced by increase of odontode rows, in crown (L, K), posterior (M) and basal (J) view. (N) Longitudinally sectioned trunk scale (IVPP V 11949.10) showing the relationships between secondary and primary odontodes. (O) Transversely sectioned trunk scale (IVPP V 11949.11), not depicted in full, showing the triangular cross-section of primary odontodes and orientation of fibre-bundles in the base. Arrowhead points at a contact between primary odontodes. Anterior to the left in (F), (N) and towards the bottom in (A), (B), (D), (E), (G–M). SEM micrographs (A–D, G–M), volume renderings (E, F) and Nomarski DIC optics micrographs (N, O).

2000 *Shiqianolepis hollandi* Sansom et al., Figs 4–6 [5].

2016 *Shiqianolepis hollandi* Andreev et al., Figs 4a–4c, 5n, 8f, 9b [7].

Holotype. Isolated trunk scale (NIGP 130294) from the Telychian Xiushan Formation, Guizhou Province, China ([5], Fig 4c and 4d).

Locality and horizon. Lower member of the Ymogantau Formation, Bachu County, Xinjiang Uygur Autonomous Region.

Material. Two isolated trunk scales and one isolated head scale (IVPP V 11951.1–3).

### Description

The trunk scales have rhombic to ovate crowns (Fig 4A–4D) with a narrow neck transitioning to a flared scale base (Fig 4E). The flattened crown has principal odontodes exposed and arranged in ridge-like rows covered by denticulate ornament (Fig 4A, 4C and 4D). Deeply incised furrows divide the principle odontode rows and these converge to meet a field of tubercles capping the secondary odontodes at the anterior crown margin (Fig 4A and 4C). The underside of the crown is without ornament and shows a number of pores (Fig 4B). The base is slightly excavated and noticeably smaller than the crown (Fig 4B).

Remarks. The correspondence in gross morphology and crown architecture between the Tarim specimens and the *Shiqianolepis* type material [5] is sufficient to regard them as conspecific.

Genus *Xinjiangichthys* Wang, Zhang, Wang & Zhu 1998 [3] sensu [7]

*Xinjiangichthys pluridentatus* Wang, Zhang, Wang & Zhu 1998 [3]

(Fig 4F–4N)

1998 *Xinjiangichthys pluridentatus* Wang, Zhang, Wang and Zhu, pl. 1, Fig A–D [3]. 1998 *Xinjiangichthys tarimensis* Wang, Zhang, Wang and Zhu, pl. 1, Fig E–I [3].

2000 *Xinjiangichthys* sp. Sansom, Aldridge and Smith, Fig 8 [5].

2016 *Xinjiangichthys pluridentatus* Andreev et al., Figs 4D–F, 6M, 8G–H [7].

Holotype. Isolated trunk scale (IVPP V 11663.1) from the Ymogantau Formation, Xinjiang, China ([3], Pl. IA, B).

Material. Circa forty isolated trunk scales, including figured specimens (IVPP V 11664.3–9).

Locality and horizon. Lower and upper members of the Ymogantau Formation, Bachu County, Xinjiang Uygur Autonomous Region.

### Description

Scale crowns are trapezoid to rhombic (Fig 4F–4I, 4K–4N) with a well-defined neck and a pronounced posterior curvature in lateral profile (Fig 4J). They consist of numerous needle-like odontodes arranged in closely packed rows, whose lower surface is pitted by pores in proximity the crown neck (Fig 4H). The base is irregular-shaped to rhombic with a concave profile (Fig 4H and 4L).

Remarks. Andreev et al. [7] synonymized the *Xinjiangichthys* type material [3] with “*Xinjiangichthys* sp.” from the Xiushan Formation (Shiqian County, Guizhou Province, [5]) and recognized *X*. *pluridentatus* as the only valid species of the genus. The Ymogantau Formation specimens illustrated here (Fig 4F–4N) similarly fall within the morphological range of *X*. *pluridentatus* by demonstrating its diagnostic features; smooth, needle-shaped odontodes arranged in rows, pronounced crown neck.

Genus *Tielikewatielepis* gen. nov.

Derivation of name. After Tielikewatie, the type Silurian section of Kalpin County, and *lepis*, Greek for scale.

Type species. *Tielikewatielepis sinensis* gen. et sp. nov.

Diagnosis. As for type species.

*Tielikewatielepis sinensis* gen. et sp. nov.

(Fig 5)

Derivation of name. *Sinensis* (Greek), pertaining to China

Holotype. An isolated trunk scale, IVPP V 11950.10 (Fig 5A).

Material. Hundreds of isolated trunk and head scales as well as six thin-sectioned trunk scales, including figured specimens (IVPP V 11950.1, 5, 6, 11–14).

Locality and horizon. Upper Member of the Tataertag Formation, Kalpin County and lower and upper members of the Ymogantau Formation, Bachu County, Xinjiang Uygur Autonomous Region.

Diagnosis. Mongolepid fish whose mature trunk scales develop arrowhead-shaped odontodes arranged in one medial and a pair or pairs of lateral arrays that diverge towards the crown’s posterior.

### Description

Morphology. Head scales are distinguished by absence of a clear separation of crown and base (Fig 5L). They are low profile elements with an antero-posterior polarity indicated by diverging rows of arrowhead-shaped odontodes. Scale bases have a flattened appearance and extend beyond the crown perimeter.

Trunk scales possess rhombic crowns with a pronounced neck developed along the contact with the base (Fig 5A–5K). The crowns display arrowhead-shaped primary odontodes arranged in, presumably, mature specimens in a pair or pairs of rows oriented at an angle to the principle medial row. These flanking rows diverge posteriorly and quickly lose contact due to deepening of the furrows between them (Fig 5A, 5D and 5E). Lanceolate secondary odontodes (Fig 5A, 5D and 5H) with a strong central ridge form along the crown’s anterior and these partially overlap the oldest portions of the main odontocomplexes. The sub-crown surface bears no ornament but is marked by discontinuous longitudinal furrows leading to pore-like openings (Fig 5C). The crown neck flares out to extend over the entire upper portion of the basal tissue leaving exposed only its smooth lower surface. The latter has a slightly convex to bulbous profile (Fig 5F, 5G, 5J and 5I) and margins generally conforming to the outlines of the crown.

Histology. Scale tissues manifest a lack of large vascular spaces/canals, including absence of distinct pulp cavities within crown odontodes (Fig 5M and 5N). The sole component of the odontodes is atubular dentine (lamellin) with lamellar and globular patterns of mineralisation (Fig 5N). Along the length of odontocomplex rows odontode height grows in posterior direction whilst uninterrupted contact between odontodes is maintained at their overlap.

The basal bone harbours flattened cell lacunae within a lamellar matrix characterized by parallel fibre spaces that propagate apically through the tissue (Fig 5M and 5N).

Remarks. *Tielikewatielepis* gen. nov. crown architecture closely matches the diverging odontode rows described in *Shiqianolepis* [5, 7], but its arrowhead-shaped odontodes and absence of tuberculate ornament separate it from any of the known mongolepid taxa.

Genus *Xiaohaizilepis* gen. nov.

Derivation of name. From Xiaohaizi, the section from which the type material was collected, and *lepis*, Greek for scale.

Type species. *Xiaohaizilepis liui* sp. nov.

Diagnosis. As for type and only species.

*Xiaohaizilepis liui* gen. et sp. nov.

(Fig 6)

1996 Chondrichthyes indet. Wang et al., Pl. II 4a, b, 5a, b [10].

Derivation of name. From ‘liui’, recognizing the contribution of Professor Liu Hsienting to the study of Chinese fossil fish.

Holotype. An isolated trunk scale, IVPP V 11949.1 (Fig 6H and 6I).

Material. Hundreds of isolated trunk scales and nine thin-sectioned scales, including figured specimens (IVPP V 11949. 1–5, 9–11).

Locality and horizon. The Tataertag Formation and the lower member of the Ymogantau Formation at Bachu County, Xinjiang Uygur Autonomous Region.

Diagnosis. Mongolepids with trunk scales possessing primary odontodes exposed on the crown surface as elongate, reclined elements oriented parallel to each other. Odontodes possess an anteriorly bifurcated medial ridge running along their length and neck canal openings at the crown’s posterior margin.

### Description

Morphology. Trunk-type scales have well-delineated crowns with more or less rhomboidal outlines (Fig 6A, 6B, 6D–6L). The main constituents of the crown are primary odontodes organized into sub-parallel rows within which the anterior most elements are those with the greatest surface exposure. A conspicuous medial ridge which bifurcates close to the anterior crown margin is a prominent feature of the primary odontodes. Diminutive pyramidal secondary odontodes (Fig 6A, 6I and 6L) are commonly found on the anterior crown margin, and are particularly numerous in ontogenetically mature specimens.

The sub-crown surface bears grooves leading to gaps between odontocomplexes (Fig 6B, 6G and 6H) at the point of their separation at the posterior of the crown. The crown/base transition exhibits a pronounced constriction (neck) with a series of horizontally distributed openings (Fig 6M). The scale base is rhombic in shape with a central protuberance.

Histology. Crown odontodes are formed of lamellin-type tissue with globular as well as lamellar texture (Fig 6N), especially prominent in the mineralisation lines around the rudimentary pulp cavities. The anterior crown margin bears wedge-like secondary odontodes that overlap the ends of primary odontocomplex rows, each composed of several odontode generations.

The basal bone of scales harbours compressed cell lacunae aligned to the tissue’s lamellae (Fig 6N and 6O). Fibre spaces penetrate the thickness of the bony base, which shows little evidence for the presence of vascular canals.

Remarks. When compared to other taxa within the Shiqianolepidae, *Xiaohaizilepis liui* shows a suite of odontode characteristics (elongate shape, bifurcating ridges and organization into a series of parallel rows) that justify the erection of a new taxon.

*Chenolepis* Sansom et al. 2000

Type and only species. *Chenolepis asketa* reported from the Xiushan [5] and Ymogantau Formations (this study) of China.

Diagnosis. As for the type species.

*Chenolepis asketa* Sansom et al. 2000 [5].

(Fig 7)

2000 *Chenolepis asketa* Sansom, Aldridge and Smith, Fig 13 [5].

Holotype. Isolated trunk scale (NIGP 130332) from the Telychian Xiushan Formation, Guizhou Province, China ([5], Fig 13b).

Emended diagnosis. Mongolepid fish possessing trunk scales with radiating rows of smooth odontodes that apically form a prominent conical cusp. The anterior of the crown is marked by the tuberculate ornament of secondary odontodes.

Material. Twenty-four isolated trunk scales and one thin-sectioned scale, including figured specimens (IVPP V 13773.1–5).

Locality and horizon. Ymogantau Formation, Kalpin County, Xinjiang Uygur Autonomous Region.

**Fig 7:**
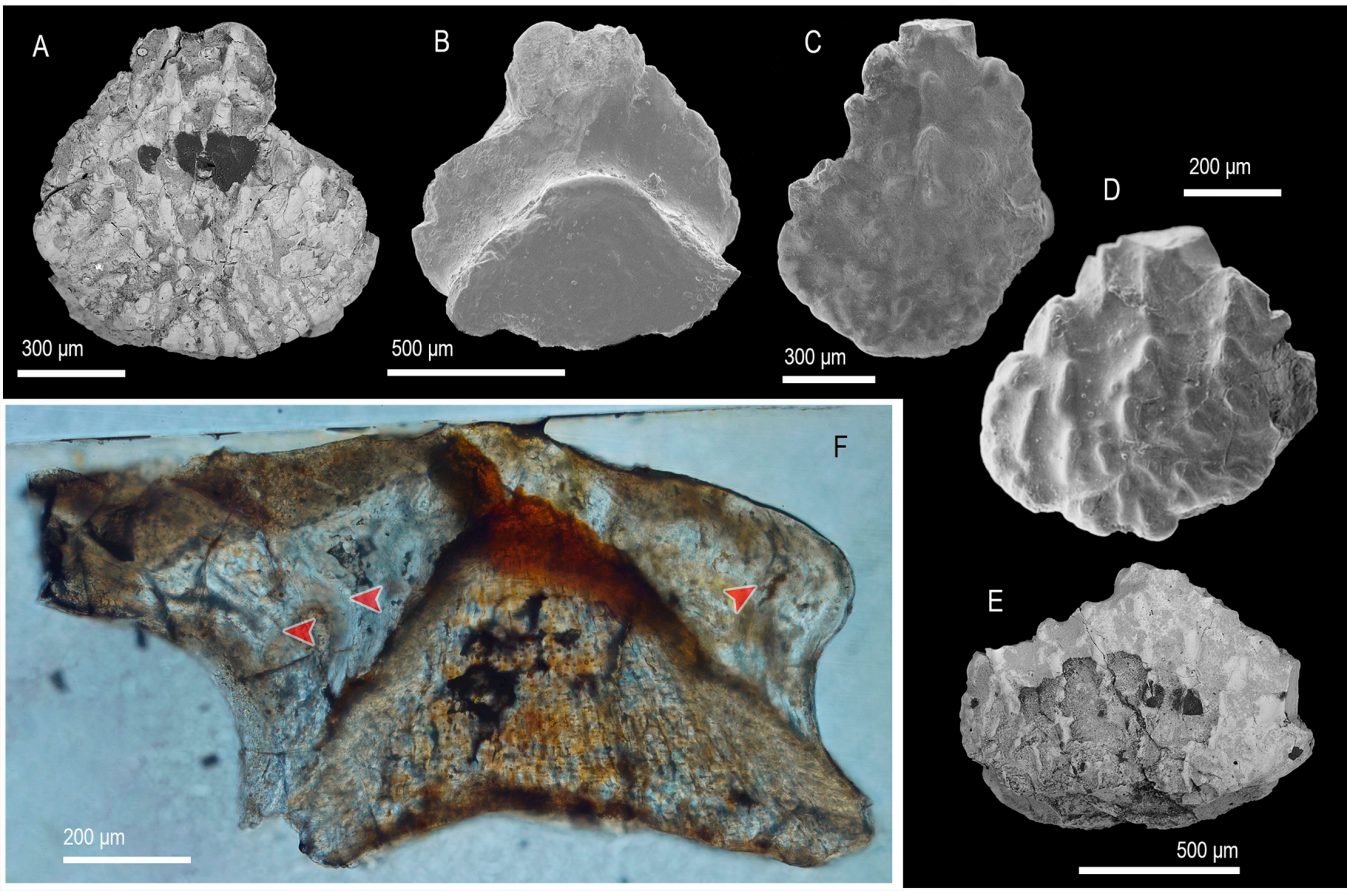
Scale morphology and histology of *Chenolepis asketa* Sansom, Aldridge and Smith 2000 [5]. (A, B) Trunk scale (IVPP V 13773.1) demonstrating the typical for the genus cuspidate primary odontodes, in crown (A) and basal (B) view. (C) Trunk scale (IVPP V 13773.2) with an elongated crown, in crown view. (D) Trunk scale (IVPP V 13773.3) with small number of odontode rows in crown view. (E) Trunk scale (IVPP V 13773.4) with a broad crown in crown view. (F) Longitudinally sectioned trunk scale (IVPP V 13773.5) showing a crown composed of lamellin. Arrowheads point at contacts between primary or secondary odontodes. Anterior to the right in (F) and towards the bottom in (A–E). SEM micrographs (A–E) and a Nomarski DIC optics micrograph (F).

### Description

Morphology. Individual scales have an ovate to oblong appearance (Fig 7A–7E) and demonstrate rows of odontodes diverging from a point near the anterior of the crown. The odontodes are reclined posteriorly and at their tip develop a prominent conical cusp that issues from the main body of each element (Fig 7A, 7C–7E and 7F). In the posterior odontodes, this cusp is seen flanked by several pairs of accessory ‘cusplets’ (Fig 7A). Wedge-shaped secondary odontodes (Fig 7A, 7C and 7D), with an ornament of tubercles, form the crown anterior to the primary odontode rows. The crown attaches via a well-defined neck to an anteriorly offset base; the latter is polygonal to elliptical and possesses a slightly hollowed lower surface (Fig 7B).

Histology. The scale crown is constructed of atubular dentine demonstrating the calcospherites and scalloped mineralisation lines of lamellin (Fig 7F). In longitudinal section the posterior generations of primary odontodes are seen to contain vestiges of pulp cavities that are closed off in the smaller secondary odontodes. The basal bone possesses fusiform cell lacunae embedded in a matrix of slightly arched lamellae of fibre bundles following the outline of the lower base surface (Fig 7F). The bone tissue is penetrated by vertical fibre spaces that converge apically.

Remarks. The new data on scale architecture and histology of *Chenolepis* reveal its identity as a mongolepid, specifically a member of the Shiqianolepidae. Another characteristic not observed by Sansom et al. [5], due to matrix adhering to the surface of the Xiushan specimens, is the unusual morphology of *Chenolepis* odontodes. These do not taper gently to a point, as for example seen in *Xinjiangichthys* ([3, 5], this study), but develop an apical cusp out of the main odontode body.

Order Sinacanthida ordo nov.

Included families. Sinacanthidae Zhu, 1998 [4].

Diagnosis. As for Sinacanthidae sensu Sansom et al. (2000) [5].

Remarks. The new taxon introduces parity of taxonomic rank between scale-based (Mongolepidida) and spine-based (Sinacanthida ordo nov.) groupings of lamellin forming chondrichthyans.

Family Sinacanthidae Zhu, 1998 [4]

Spine morphology A

(Fig 8A–8D and 8K)

Material. Multiple spine fragments, including figured specimens (IVPP V 13774.1–5).

Locality and horizon. Ymogantau Formation, Bachu County, Xinjiang Uygur Autonomous Region.

**Fig 8:**
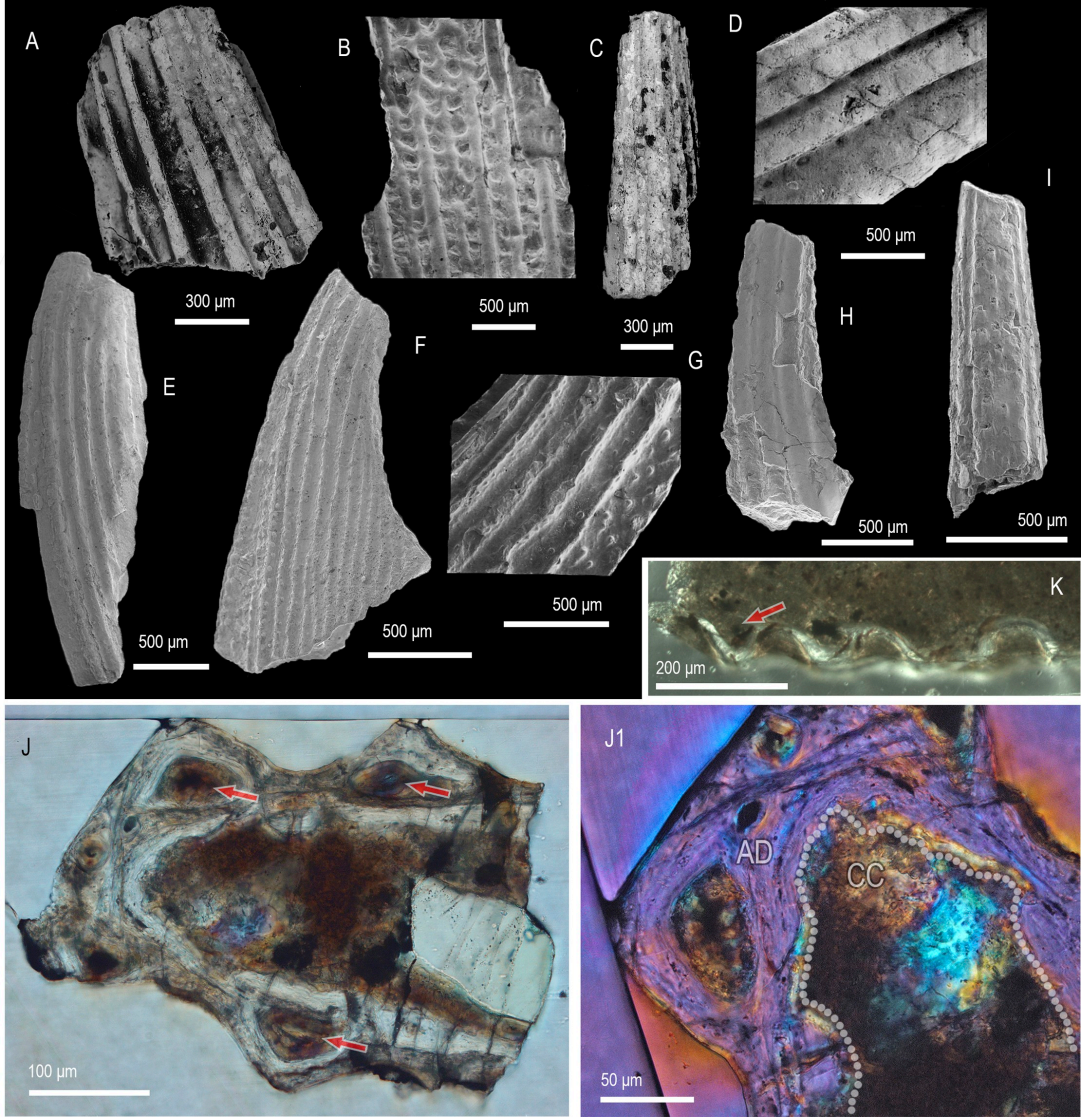
Morphology and histology of sinacanthid spines. **(**A–D, K) Spine morphology A. (A) Lateral wall of a spine fragment (IVPP V 13774.1) showing the width and distribution of ornamenting ridges, lateral view. (B) Supposed basal fragment of a spine (IVPP V 13774.2) showing strong segmentation of ridges, lateral view. (C) Apical portion of a partial spine (IVPP V 13774.3), anterior lateral view. (D) Spine fragment (IVPP V 13774.4) demonstrating the rhombic secondary ornament of ridges. (E–I) Spine morphology B. (E) Apical spine fragment (IVPP V 13775.1) in lateral view. (F) Incomplete spine (IVPP V 13775.2) demonstrating the characteristic for the type strong keel and narrow ornamenting ridges, lateral view. (G) Detailed depiction of the main ornamenting ridges and the tuberculate ornament of the keel of an incomplete spine (IVPP V 13775.3), lateral view. (H) Apical fragment of a spine (IVPP V 13775.4) in lateral view. (I) Apical fragment of spine (IVPP V 13775.5) showing a nodose ornament along its anterior edge. (J) Transversely sectioned spine (IVPP V 13775.6) demonstrating the atubular dentine of the spine trunk and formation of large denteons (arrows) inside the ornamenting ridges. (J1) Detailed view of (J) showing the relationship between the lamellar dentine and the calcified cartilage formed inside the spine’s central cavity. (K) Part of a lateral wall of a transversely sectioned spine fragment (IVPP V 13774.5). AD, atubular dentine, CC, calcified cartilage. Dotted line marks the lamellar dentine/calcified cartilage boundary. Arrows point at denteons in J and K. Anterior to the left in (C, E, F, H, J, K), to the right in (A, B, G) and towards the top in (J1). SEM micrographs (A–I) and Nomarski DIC optics micrographs (J, J1). (K).

### Description

Morphology. Spines carry a strong ornament of flat-topped ridges with corrugated margins that in part are divided into rhombic/lanceolate segments (Fig 8A–8D). Ridges exhibit subparallel orientation and uneven spacing on the flattened lateral sides of spines (Fig 8A). The spines have a narrow cross section with a shallow posterior indentation representing the sulcus of the posterior edge.

Histology. The tissue structure of sectioned specimens can be discerned in the ridges of the spine ornament that comprise of atubular dentine formed around a central canal (Fig 8K).

Remarks. The spines’ atubular dentine (Fig 8K), boxy profile of the ridge ornament and notable lateral compression support assignment to the Sinacanthidae, following Sansom et al. [5],[6]. The fragmentary nature of the material, however, precludes determination of spine shape and ridge count that are used to determine sinacanthid morphotaxa [4].

Spine morphology B

(Fig 8E–8J1)

Material. Seven incomplete spines and multiple spine fragments, including figured specimens (IVPP V 13775.1–6).

Locality and horizon. Ymogantau Formation, Bachu County, Xinjiang Uygur Autonomous Region.

### Description

Morphology. Recurved spines that broaden in their profile at the base (Fig 8F). Spine surfaces bear evenly spaced subparallel ridges with triangular cross section and corrugated margins (Fig 8E–8H, 8J and 8J1). The ridges break down into tuberculate/nodose ornament along the anterior edge (Fig 8F, 8G and 8I) where the spine develops a laterally compressed keel that widens towards the base (Fig 8F). The posterior spine margin is marked by a deep sulcus (Fig 8E).

Histology. Spines consist of a type of atubular lamellar dentine with traces of calcospheritic mineralisation, lined internally by an optically less distinct globular calcified cartilage (Fig 8J and 8J1). The centre of spine ridges is occupied by a large denteon distinguished by concentric lamellae formed around a vascular canal (Fig 8J and 8J1). The denteon tissue extends into the inter-ridge spaces in a continuous manner around the spine’s perimeter. On its inner surface the dentine is bounded by remnants of optically faint calcified cartilage with globular microstructure (*sensu* [48]), evidenced by mineralised spherites and wavy precipitation lines (Liesegang waves).

Remarks. The histological signature of sinacanthid spines, an outer sculpted layer of atubular dentine and an inner layer of globular calcified cartilage ([6]), is also found in the morphology B spines. A notable feature of morphology B is the lamellin-like appearance of the dentine tissue, with a mineralisation pattern akin to that of mongolepid scale crowns [5, 7, 49]. In *Sinacanthus*, and likely in *Neosinacanthus* and *Tarimacanthus*, the dentine layer has a distinctly globular texture inside the ridges and becomes lamellar only interior of the ornament [4, 6]. Other characteristics of morphology B spines not reported previously in sinacanthids are the development of a strong keel and ridges with a triangular cross section.

In the absence of articulated material, it is unclear what portion of the taxa assigned to Sinacanthida ordo nov. need to be synonymized, given that a number of more completely known stem-group chondrichthyans show a similar range of spine morphologies within individual specimens (e.g. *Parexus recurvus*, *Climatius reticulatus* and *Doliodus problematicus* [50–52]). With this in mind, and in order to avoid creating a series of sinacanthid morphotaxa, we have adopted open nomenclature for the spines described here.

Order incertae sedis

Family incertae sedis

Genus *Yuanolepis* gen. nov.

Derivation of name. After Professor F.L. Yuan, one of the first geologists to study the early vertebrates of Xinjiang, and *lepis*, Greek for scale.

Type and only species. *Yuanolepis bachunensis* gen. et sp. nov.

Diagnosis. As for type species.

*Yuanolepis bachunensis* gen. et sp. nov.

(Fig 9)

Derivation of name. After the Bachu fossil locality.

**Fig 9:**
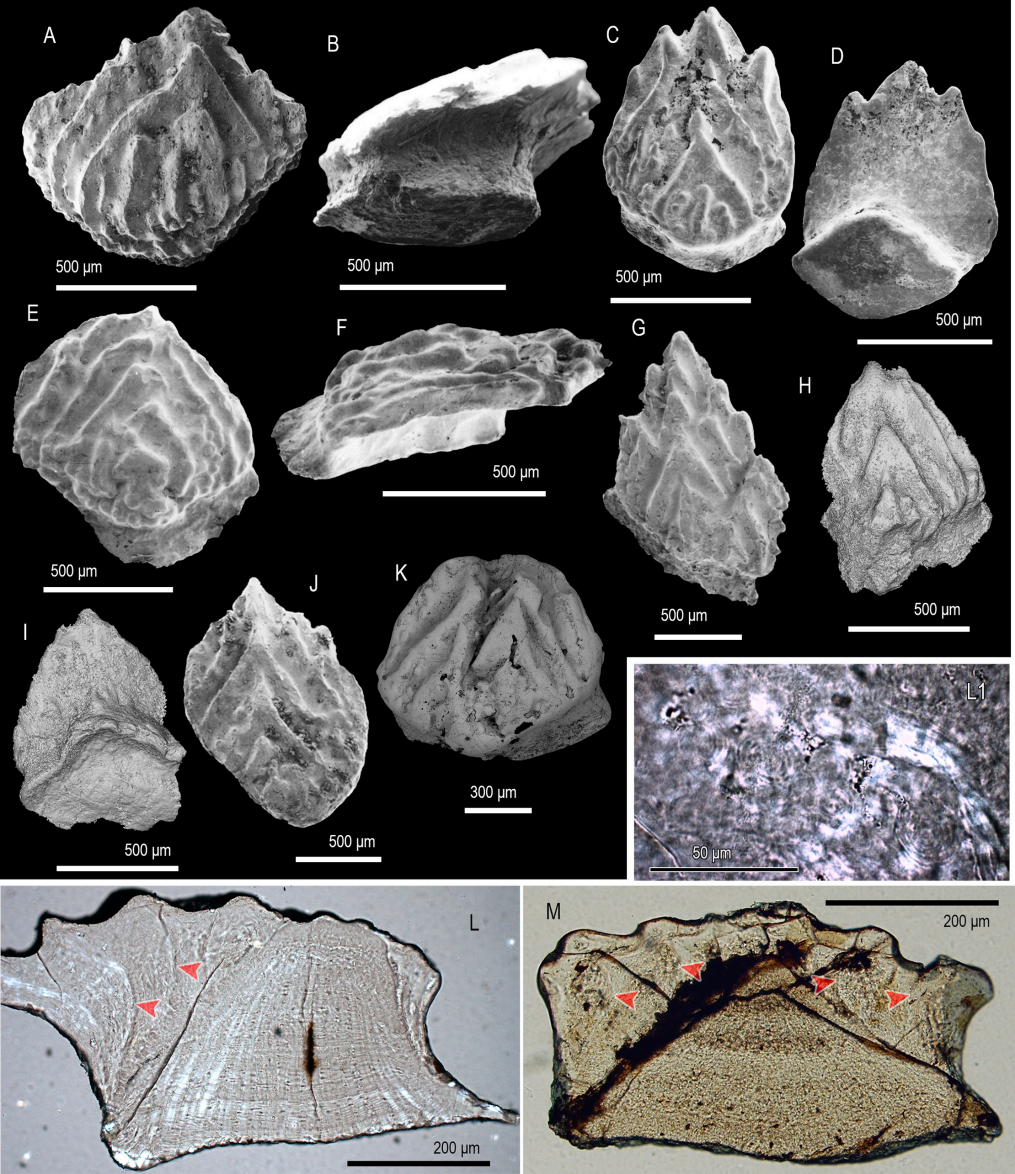
Scale morphology and histology of *Yuanolepis bachunensis* gen. et sp. nov. (A, B) Trunk scale (IVPP V 17709.1) at an early stage of development in crown (A) and lateral view (B), holotype. (C, D) Trunk scale (IVPP V 17709.2) with an elongated crown, in crown (C) and basal (D) view. (E, F) Trunk scale (IVPP V 17709.3) at late stage of development, in crown (E) and lateral (F) view. (G) Asymmetrical trunk scale (IVPP V 17709.6) in crown view. (H, I) Trunk scale at early stage of development (IVPP V 17709.7) in crown (H) and basal (I) view. (J) Elongated trunk scale (IVPP V 17709.8) in crown view. (K) Trunk scale (IVPP V 17709.9) with a broad crown in anterior crown view. (L) Longitudinal section of a trunk scale (IVPPV17709.4) demonstrating the arrangement of primary of secondary odontodes, shown in part. (L1) Detail of (L) showing the structure of lamellin at the posterior of the crown. (M) Transverse section of a trunk scale (IVPP V 17709.10). Arrowheads point at contacts between primary odontodes. Anterior to the left in (B, F), to the right in (L, L1) and towards the bottom in (A–E, G–K). SEM micrographs (A–G, J, K), volume renderings (H, I) and Nomarski DIC optics micrographs (L, L1, M).

Holotype. Isolated trunk scale, IVPP V 17709.1 (Fig 9A–9D).

Material. Seventeen isolated trunk scales and three thin sectioned scales, including figured specimens (IVPP V 17709.2–4, 6–10).

Locality and horizon. Ymogantau Formation in Bachu County and the lower member of the Ymogantau Formation in Kalpin County, Xinjiang Uygur Autonomous Region.

Diagnosis. A species possessing trunk scales with overlapping deltoid odontodes that carry ridged and tuberculate ornament. Odontodes consist of atubular dentine and arrange in a single row where they become larger posteriorly.

### Description

Morphology. All specimens display scale crowns with a neck-like constriction and an antero-posterior polarity (Fig 9A–9L). The crowns are elliptical to deltoid with serrated/corrugated posterior margins. Primary crown odontodes bear strong ridges and tubercles along their periphery. On the crown surface they appear as deltoid/elliptical overlapping elements deposited in a growth series (odontocomplex), with smaller heavily ornamented secondary odontodes forming along the crown’s anterior (Fig 9A, 9C and 9J). The sub crown is smooth and devoid of canal openings. Scale bases are rhombic with a slight central protrusion (Fig 9D and 9I).

Histology. The crown’s primordial odontode sits at the apex of the base, being the smallest component of a primary odontocomplex within which odontode size increases towards the posterior (Fig 9L). Odontodes consist of an atubular dentine tissue demonstrating extensive globular mineralisation and absence of clearly recognizable pulp cavity spaces (Fig 9L, 9L1 and 9M). The basal bone has a distinctly lamellar appearance with spindle-shaped cell spaces distributed throughout the tissue (Fig 9L and 9M). Spaces for fibre bundles run across the thickness of the base in a subparallel manner, assuming slightly undulating trajectories along their course (Fig 9L and 9M).

Remarks. *Yuanolepis* gen. nov. scale crowns have a lamellin-like histology but are readily distinguished from known mongolepid species in form and structure [5, 7]. Instead of the multiple odontode rows characteristic of Mongolepidida [7], *Yuanolepis* gen. nov. have single-odontocomplex scales with an appositional growth pattern. This crown architecture occurs in the Chondrichthyes *sensu lato* [24, 53] within ‘acanthodians’ and euchondrichthyans (e.g. *Seretolepis*, *Kathemacanthus*, *Parexus*, *Wodnika* [24, 50, 54, 55]) and is indicative of affinity to the clade. The overall geometry of the crown in these specimens suggests they are trunk scales.

## Discussion

**Fig 10:**
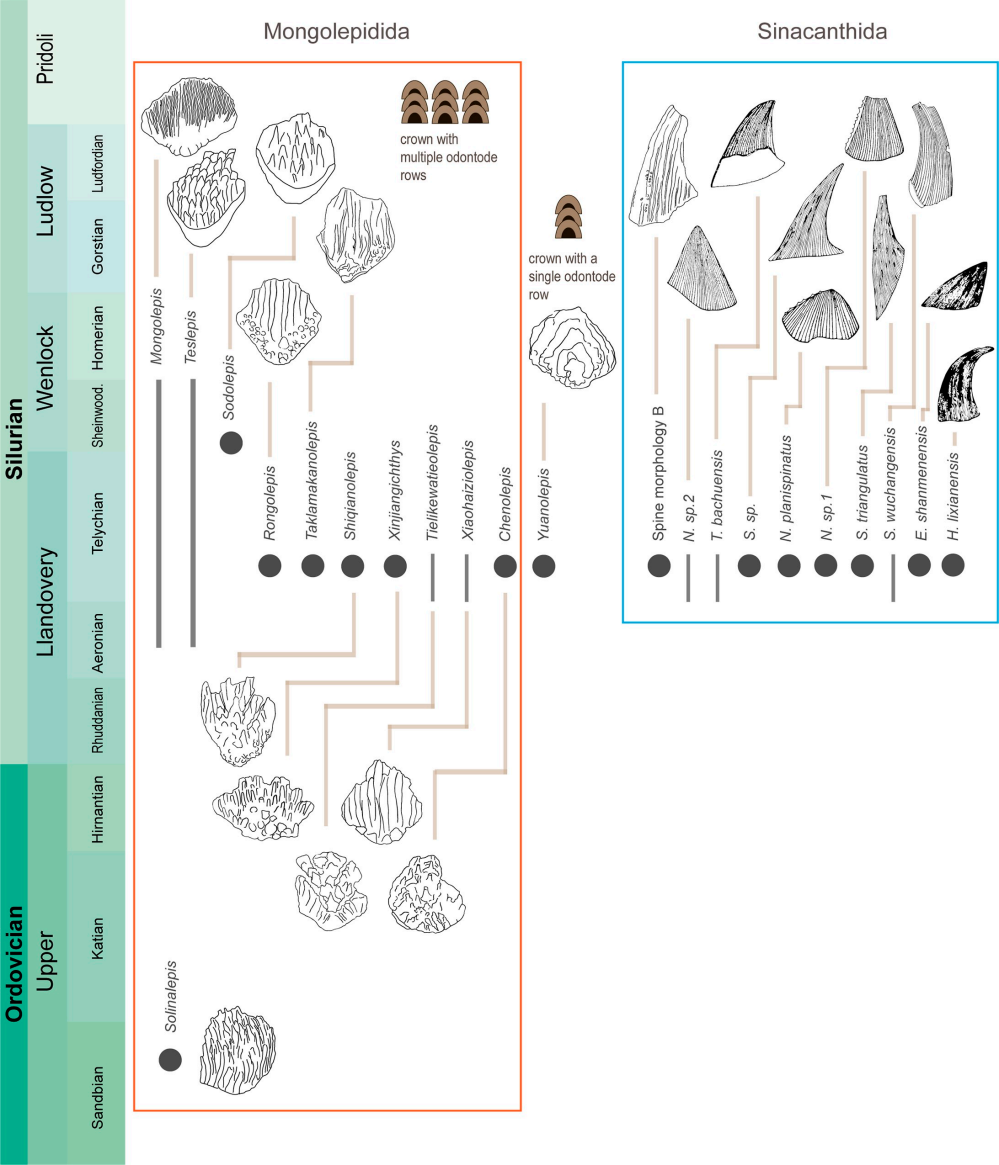
Stratigraphic ranges of lamellin-forming chondrichthyans. Diagram of all formally described mongolepids, *Yuanolepis* gen. nov. and sinacanthids (based on data from [2, 4, 5, 7, 8], this study). Ranges of taxa represented by dark grey bars and circles. Line art of sinacanthid spines modified from [2, 4]. Abbreviations of generic names: *E*. = *Eosinacanthus*, *H*. = *Hunanacanthus*, *N*. = *Neosinacanthus*, *S*. = *Sinacanthus*, *T*. = *Tarimacanthus*.

The Xinjiang taxa described here expand the Mongolepidida to eleven formally described genera, adding to records from North America, Mongolia and South China [3, 5, 7, 18, 49, 56, 57] (Fig 10). The mongolepids are the most widely distributed (in stratigraphic and palaeogeographic senses) scale-based components of the earliest chondrichthyan faunas, with microvertebrate assemblages from the Siberian Platform hinting that their diversity in the Silurian (Llandovery–Wenlock) might be greater than currently recognised [19, 58]. The material from the Tataertag and Ymogantau Formations has extended the overlap between the Tarim and South China constituents of the Zhangjiajie Vertebrate Fauna. These data further underscore the impoverished nature of the Zhangjiajie Fauna (mongolepids, sinacanthids and galeaspids) when compared with coeval sites from Siberia and Mongolia and their complement of mongolepids, acanthodians, thelodonts, eriptychiids and heterostracans [19, 59].

As far as can be determined, all Chinese mongolepids come from Telychian vertebrate assemblages (from the Xiushan, Tataertag and Ymogantau Formations) where they co-occur with a variety of sinacanthid spines ([8] and references therein; this study). This has prompted some authors to suggest grouping together mongolepids and sinacanthids on the basis of shared atubular dentine and the absence of other associated chondrichthyan-like remains [60]. The presence of mongolepid scales in other sinacanthid-bearing Formations (such as the Rongxi and Fentou from South China) is yet to be determined as these have not been sampled for microremains [2, 8], and the abundance of associated sinacanthid fossils is not a feature of Mongolian and North American mongolepid assemblages [18, 57, 58]. In the absence of definitive proof for a co-association of mongolepid scales and sinacanthid spines, the discovery of *Yuanolepis* gen. nov. has bearing on the affinities of the Mongolepidida and their relationship to the Sinacanthidae. Although falling outside of the current definition of a mongolepid, developmentally the scales of *Yuanolepis* gen. nov. are distinctly chondrichthyan (see Remarks). It may be reasonable to suggest an expanded grouping for lamellin-forming taxa including mongolepids and taxa with the *Yuanolepis* gen. nov. type scale crown architecture (Fig 10). Their single- and poly-odontocomplex patterns of odontode addition also appear in more derived components of the chondrichthyan stem (e.g. the *Ctenacanthus* and *Seretolepis* morphogenesis types *sensu* [24]), in non-acanthodid taxa (*sensu* [30]). The arrays of paired spines possessed by a number of these species (e.g. *Doliodus*, *Kathemacanthus*, *Climatius* and *Parexus* [50–52, 55, 61]) show remarkable similarity to sinacanthid morphological types (Fig 10), whilst being constructed primarily of tubular osteodentine [50–52, 55, 61]. Significantly, the *Seretolepis*-like architecture of *Yuanolepis* gen. nov. scales (see Remarks) is present in the same region of the stem, among members of the now paraphyletic Climatiiformes (e.g. *Brochoadmones*, *Seretolepis*, *Kathemacanthus*, *Climatius and Parexus* [50, 51, 55, 62–64]). This leads us to view lamellin-forming chondrichthyans as possessing modes of morphogenesis previously recorded only in a subset of stem chondrichthyans. Our present understanding of the former points towards them being on a branch supporting the euchondrichthyan node, crownward of ‘acanthodians’ retaining the plesiomorphic box-in-box mechanism of odontode addition [26, 27, 30, 65, 66].
